# Synovial fluid mesenchymal progenitor cells from patients with juvenile idiopathic arthritis demonstrate limited self-renewal and chondrogenesis

**DOI:** 10.1038/s41598-022-20880-7

**Published:** 2022-10-03

**Authors:** Roman J. Krawetz, Asmaa Affan, Catherine Leonard, Dwaraka Natha Veeramreddy, Akash Fichadiya, Liam Martin, Heinrike Schmeling

**Affiliations:** 1 McCaig Institute for Bone and Joint Health, Faculty of Medicine, University of Calgary, 3330 Hospital Drive NW, Calgary, AB T2N 4N1 Canada; 2 Department Cell Biology and Anatomy, University of Calgary, Calgary, AB Canada; 3 Department of Surgery, University of Calgary, Calgary, AB Canada; 4 Biomedical Engineering Graduate Program, University of Calgary, Calgary, AB Canada; 5 Department of Pediatrics, University of Calgary, Calgary, AB Canada; 6 Alberta Children’s Hospital Research Institute, University of Calgary, Calgary, AB Canada; 7 Department of Medicine, University of Calgary, Calgary, AB Canada

**Keywords:** Rheumatology, Rheumatic diseases, Mesenchymal stem cells, Regeneration

## Abstract

Juvenile idiopathic arthritis (JIA) is a heterogeneous group of inflammatory diseases affecting joints with a prevalence of one in a thousand children. There is a growing body of literature examining the use of mesenchymal stem/progenitor cells (MPCs) for the treatment of adult and childhood arthritis, however, we still lack a clear understanding of how these MPC populations are impacted by arthritic disease states and how this could influence treatment efficacy. In the current study we examined the immunophenotyping, self-renewal ability and chondrogenic capacity (in vitro and in vivo) of synovial derived MPCs from normal, JIA and RA joints. Synovial MPCs from JIA patients demonstrated reduced self-renewal ability and chondrogenic differentiation capacity. Furthermore, they did not induce cartilage regeneration when xenotransplanted in a mouse cartilage injury model. Synovial MPCs from JIA patients are functionally compromised compared to MPCs from normal and/or RA joints. The molecular mechanisms behind this loss of function remain elusive. Further study is required to see if these cells can be re-functionalized and used in cell therapy strategies for these JIA patients, or if allogenic approaches should be considered.

## Introduction

Juvenile idiopathic arthritis (JIA) is the most common chronic childhood rheumatic disease with an estimated incidence of one in a thousand (~ 25,000 Canadian children^1^)^2,3^. Early diagnosis and treatment of JIA is essential to achieve optimal patient outcomes and while patient outcomes of JIA have improved over the last decade, many still face the possibility of continuing disease activity, medication-associated morbidity and life-long disability^4^. The symptoms of this disease (pain, limited range of motion) have the potential to significantly impact the quality of life, academic/athletic performance, and peer relationships in children with JIA^5^. Therefore, it is essential to better understand the mechanisms involved with disease severity and progression in addition to developing new therapies that treat the disease instead of solely mitigating symptoms.

Many groups are examining the use of cellular therapies, such as mesenchymal stem/progenitor cells (MPCs) to repair cartilage or modulate inflammation to promote healing. However, little efficacy in promoting cartilage repair, or reducing patient symptoms over current surgical treatments such as micro-fracture has been observed^6–8^. There are only a very few instances of MPCs being used in JIA patients, but in these limited reports, beneficial outcomes have been observed^9–11^. In a case series of JIA patients (3 children) that were given MPCs (derived from adipose, umbilical or Wharton’s Jelly) and all three tapered off conventional immunosuppressive drugs with no serious adverse events^10^. In another study, JIA patients (6 children) were given bone marrow MPCs with variable outcomes observed^9^. While 9 patients are not sufficient to gauge efficacy, it does show that MPCs deserve further study as a potential treatment of JIA.

There is a growing body of literature demonstrating that MPCs derived from the synovial lining of the joint are superior in terms of chondrogenic differentiation^12–14^. These MPCs also share a common developmental origin with the articular cartilage^15^ and have been demonstrated to show a bias towards chondrogenesis vs. osteogenesis or adipogenesis^14,16^. These synovial MPCs also demonstrate robust immunomodulation capabilities^17,18^ that can be disrupted by chronic inflammation (such as in RA patients^19^), during joint degeneration^20^ and/or the amount of synovial fluid in the joint^21^. These findings are relevant to JIA, as cell therapy may be a promising treatment option for JIA patients. Therefore, it is essential to understand if, how and why stem/progenitor cell function is altered in patients with JIA. Furthermore, it is widely accepted that once a patient is diagnosed with arthritis, it may only be a matter of time before some level of invasive intervention is required to moderate the symptoms (pain, loss of function and mobility) associated with disease progression^2,3^. Understanding the role of endogenous stem/progenitor cells in the apparent lack of cartilage homeostasis and/or repair is paramount in developing novel therapeutics to restore the repair capacity of these cells, and ultimately heal damaged tissue. A logical first step is to identify if and how the injured and/or early arthritic joint environment impacts endogenous MPCs. Therefore, in the current study, we isolated MPCs from synovial fluid from normal, RA and JIA patients and compared their chondrogenic differentiation capacity in vitro and in vivo.

## Methods

### Subjects

Patients with clinically diagnosed JIA (Oligoarticular Persistent/Extended; RF negative Polyarticular) had synovial fluid aspirated from their knee(s) (mean volume 18.3 ml + / − 16.2 ml) during a visit to the Alberta Children’s Hospital (N = 19, 6 males age: 10, 11, 16, 16, 17, 18; 13 females age: 3, 5, 5, 6, 7, 10, 11, 12, 12, 13, 13, 16, 17).

Patients with clinically diagnosed RA with no other co-morbidities had synovial fluid aspirated from their knee(s) (mean volume 43.1 ml + / − 8.3 ml) during a visit to the University of Calgary Foothills Medical Clinic (N = 5, 2 males age: 46, 55; 3 females age 47, 55, 59)^22–24^.

Synovial fluid from macroscopically normal knees was aspirated (mean volume 5.6 ml + / − 2.2 ml) from cadavers less than 4 h after death (N = 5, 2 males age: 47, 54; 3 female age 46, 51, 52). Tissue donors were received by the Southern Alberta Organ and Tissue Donation Program (SAOTDP), which obtains the medical history of every donor, including current medication, previous history of joint diseases, and other co-morbidities (e.g., cancer, diabetes, inflammatory diseases)^22,25,26^.

The JIA patients were significantly younger that the RA (p < 0.001, one way ANOVA) and normal group (p < 0.001, one way ANOVA). There was no difference in age between the RA and normal group (p = 0.6842, one way ANOVA).

### Synovial fluid MPC Culture

Primaria 6 well culture dishes (Corning) were scratched with a sterile scalpel blade to generate a roughened surface and then washed with sterile PBS prior to adding 500ul of synovial fluid to each well. The synovial fluid was left in these plates for 1–2 h at 37 °C/5%CO2 to allow for cell attachment, and then culture media was added^14,23,25,27,28^. sMPC culture media consisted of MesenCult™ (Stemcell Technologies) with 1% Pen/Strep, 1% Non-essential amino acids (NEAA) (all Invitrogen, Carlsbad, CA). Once cells had adhered to the plastic and reached 30–40% confluence, the media was changed and the cells were allowed to reach 60–70% confluence. At this point (still passage 0) all 6 wells (of each 6 well dish – 1 dish per patient) were dissociated and resuspended in Dulbecco's PBS (DPBS) at 1 million cells/ml. Primary labeled FACS antibodies to CD105, CD90, CD73, CD44, CD45, CD11b (All Becton, Dickinson and Company (BD), Franklin Lakes, NJ) were added to the suspension and the cells were sterilely sorted (University of Calgary, Flow Cytometry Core Facility) to obtain the CD105^+^CD90^+^CD73^+^CD44^+^CD45^−^CD11b^−^ population^29–33^. An aliquot of the cells was re-analyzed using flow cytometry prior to differentiation and xenotransplantation to confirm the presence of the CD105^+^CD90^+^CD73^+^CD44^+^CD45^−^CD11b^−^ population.

All MPCs were expanded to passage 15 in sMPC media using T25 CELLSTAR® culture flasks (Greiner Bio-One). For passaging, cells were scraped and split at a ratio of 1:4.

### Flow cytometry

Synovial fluid MPCs were fixed in methanol for 10 min on ice. After PBS washing, cells blocked for 30 min at 37^0^C with 3% BSA. They were then incubated away from light for 1 h with a fluorescent antibodies for CD105, CD90, CD73, CD44, CD45, CD11b (All Becton, Dickinson and Company (BD), Franklin Lakes, NJ) prior to flow cytometric analysis on an Invitrogen Attune® Acoustic Focusing Cytometer.

### Cell proliferation

The Moxi Z (ORFLO) was used to quantity MPCs numbers as well as obtain cell viability. Type M cassettes (ORFLO) with a dilution of 1:10 were employed. Each count was run twice and the result was averaged. To determine cell proliferation rates, the CyQUANT® Cell Proliferation Assay (Invitrogen) was utilized. Briefly, 10,000 cells from each treatment group were plated into 60 mm plates and samples were collected every 24 h for 8 days and the cell number was quantified using a fluorescent standard curve and micro plate reader as described in the manufactures instructions^25^.

### Differentiation

The MPCs underwent multi-lineage differentiation analysis to determine their osteo/chondro/adipo-genic capacity^30,34^.

Osteogenesis: For each replicate, 5 × 10^5^ cells were seeded into each well in a 24-well plate and then placed into DMEM/F-12 media that contained Dexamethasone (final concentration (FC): 100 nM) (Sigma), L-Ascorbic Acid (FC: 50 μg/mL) (Sigma), β-Glycerolphosphate (FC: 10 mM) (Sigma).

Adipogenesis: For each replicate, 5 × 10^5^ cells were seeded into each well in a 24-well plate and then placed into DMEM/F-12 media that contained Dexamethasone (FC: 1 μM) (Sigma), Insulin (FC: 10 μM) (Sigma), Indomethacin (FC: 200 μM) (Sigma), and Isobutylmethylxanthine (FC: 500 μM) (Sigma).

Chondrogenesis: For each replicate, 5 × 10^5^ cells were pelleted through centrifugation and placed into DMEM/F-12 media that contained Dexamethasone (**FC**: 10 nM) (Sigma), L-Ascorbic Acid (FC: 50 μg/mL) (Sigma), MEM Non-Essential Amino Acids (FC: 1%) (MEM-NEAA Gibco), Transforming growth factor (TGF)-β3 (FC: 10 ng/mL) (Peprotech), Bone morphogenetic protein (BMP)-2 (FC: 500 ng/mL) (Peprotech), insulin transferrin selenium (FC: 1%) (Lonza- BioWhittaker), and sodium pyruvate (FC: 1%) (ThermoFisher). Media was adjusted to neutral pH (7.0–7.6).

After 21 days, differentiation was assayed using reverse transcriptase quantitative polymerase chain reaction (RT-qPCR) and histological staining.

### RT-qPCR

mRNA was isolated using the TRIzol reagent protocol (ThermoFisher) following the manufactures instructions with the addition of glycogen solution (Amresco) to increase the yield of mRNA. Chondrogenic cultures alone went through an additional spin column step (OMEGA bio-tek E.Z.N.A. Total RNA Kit I) to remove additional ECM proteins which could potentially interfere with downstream applications. For first strand synthesis, mRNA was then added cDNA Master Mix (High Capacity cDNA kit, Applied Biosystems) following the manufactures instructions. The cDNA was stored at -20 °C until use.

For osteogenesis, gene expression of Osterix (*Sp7*) (Probe set # Mm00504574_m1) and *Runx2* (Probe set # Mm00501584_m1) were quantified. For adipogenesis, *Adipq* (Probe set # Mm00456425_m1) was quantified. For chondrogenesis, *Sox9* (Probe set # Mm00448840_m1) and *Col2a* (Probe set # Mm01309565_m1) were quantified. Ribosomal 18S (Probe set # Mm03928990_g1) was employed as a housekeeping gene. All TaqMan Gene Expression Assays were obtained from Applied Biosystems. Three replicates were run per sample and all samples were run on an ABI Quantstudio6 (Applied Biosystems) Resulting threshold (Ct) values were analyzed using the ΔΔCt method against 18S endogenous control and undifferentiated cells as the reference sample.

### Histological staining

For further analysis of differentiation, histological staining was undertaken. For osteogenic and adipogenic differentiations, the wells were fixed with 10% neutral buffered formalin (NBF) for one hour. The osteogenic wells were stained with a 0.2% Alizarin Red S (Sigma) solution in the dark for 10–15 min. The adipogenic wells were stained with a 0.5% Oil Red O solution (Sigma) for 15 min. For chondrogenic pellets, whole-mount staining was performed as follows. Pellets were fixed with 10% NBF for three hours, then washed with distilled water. The pellets were then stained with 1% Alcian blue in 3% acetic acid (Sigma) for 45 min in the dark. The pellets were then de-stained and transferred to PBS.

### In vivo* cartilage repair*

Animal studies were carried out in agreement with recommendations from the Canadian Council on Animal Care Guidelines and were approved by the University of Calgary Health Sciences Animal Care Committee (Ethics # ACC-2042). The reporting of this data in the manuscript follows the recommendations in the ARRIVE guidelines.

Full thickness cartilage defects (FTCD)^35–39^ were created on the tibial plateau in 12 NOD scid gamma (NOD) mice. Animals were administered an intraperitoneal injection of buprenorphine (0.05 mg/kg) prior to surgery and anaesthetized under isoflurane (Baxter) anesthesia (1.5% v/v O2) for the duration of the surgical procedure. Briefly, a small incision was made on the medial side of the left knee. A depth stopped 26G needle (diameter = 450 µm, length to stopper = 600 µm) was used to gently displace the patella and expose the trochlear groove of the femur. A slight pressure, combined with a twisting motion, was applied at the contact with the trochlear groove to make a circular wound that penetrated no farther than 600 µm into the underlying subchondral bone. The needle was gently removed, and the skin closed with a sterile wound clip after the FTCD was made.

The mice were randomly assigned to a group and intraarticularly injected 1-week post-injury with 50,000 human synovial fluid MPCs in 2 µL of sterile saline as follows^35^: Group 1: n = 3 mice with saline (controls), Group 2: n = 8 mice with MPCs from JIA patients (n = 2 mice per patient line – 4 lines used), Group 2: n = 6 mice with MPCs from RA patients (n = 2 mice per patient line – 3 lines used), and Group 4: n = 6 mice with MPCs from normal joints (n = 2 mice per patient line – 3 lines used). The mice were euthanized 4 weeks post-injection.

### Histology and immunohistochemistry

Knee joints were then fixed in 10% NBF (Fisherbrand) for 7 days and decalcified in 10% EDTA for 14 days. Samples underwent tissue processing, were embedded in paraffin wax, and then sectioned at 10 µm. Histological analysis was then conducted on the knee sections. Samples were deparaffinized in Citrosolv (Decon Laboratories), and then rehydrated in a series of ethanol washes with decreasing concentration. For histological analysis, samples were stained with Safranin-O/Fast-green and graded based on a previously published scoring system. The parameters of the scoring system include cell morphology (0–4), matrix staining (0–3), surface regularity (0–3), thickness of cartilage (0–2) and integration with native cartilage (0–2). On this scale, uninjured native articular cartilage is 14, while the absence of normal cartilage is 0^37–39^.

Immunohistochemistry analysis was performed on the knee sections. Antigen-retrieval was achieved using 10 mM sodium citrate (pH 6.0), and non-specific blocking was prevented using goat-serum (1:500 dilution in TBST). Human nuclear antigen (HNA; Clone # 235–1, Abcam) was applied to the sections and incubated overnight. Anti-rabbit secondary antibody AF647 (1:100, Biolegend) was applied the next day. Secondary controls were also performed, where only secondary antibody was applied to the sections (no primary antibody). To identify murine macrophage populations, CD38 (Clone # 90, Conjugated to AF594, Biolegend) and CD206 (Clone # C068C2, Conjugated to AF647, Biolegend) staining was undertaken using primary antibodies conjugated to fluorophores^39–41^. All slides were mounted using EverBrite™ Hardset Mounting Medium with DAPI (Biotium). Slides were imaged using a Plan-Apochromat objective (20 × /0.8 M27) on an Axio Scan.Z1 Slide Scanner microscope (Carl Zeiss; Oberkochen, Germany).

### Statistics

The RT-qPCR data were analyzed using GraphPad Prism 7 (GraphPad Software). The data had been reported as ± standard deviation (SD). Statistical analysis was performed with a paired t-test since the undifferentiated controls for each experiment performed are derived from the same parental cells as the differentiated cells. An alpha value of p < 0.05 was regarded as statistically significant.

### Ethics approval and consent to participate

Informed consent to participate was obtained by written agreement. The study protocol was approved by the University of Calgary Research Ethics Board (University of Calgary ethics # REB16-1262, REB-21987). Animal studies were carried out in agreement with recommendations from the Canadian Council on Animal Care Guidelines and were approved by the University of Calgary Health Sciences Animal Care Committee (Ethics # ACC-2042).

## Results

### JIA MPCs demonstrate decreased viability and proliferation potential with time in culture

**Figure 1:**
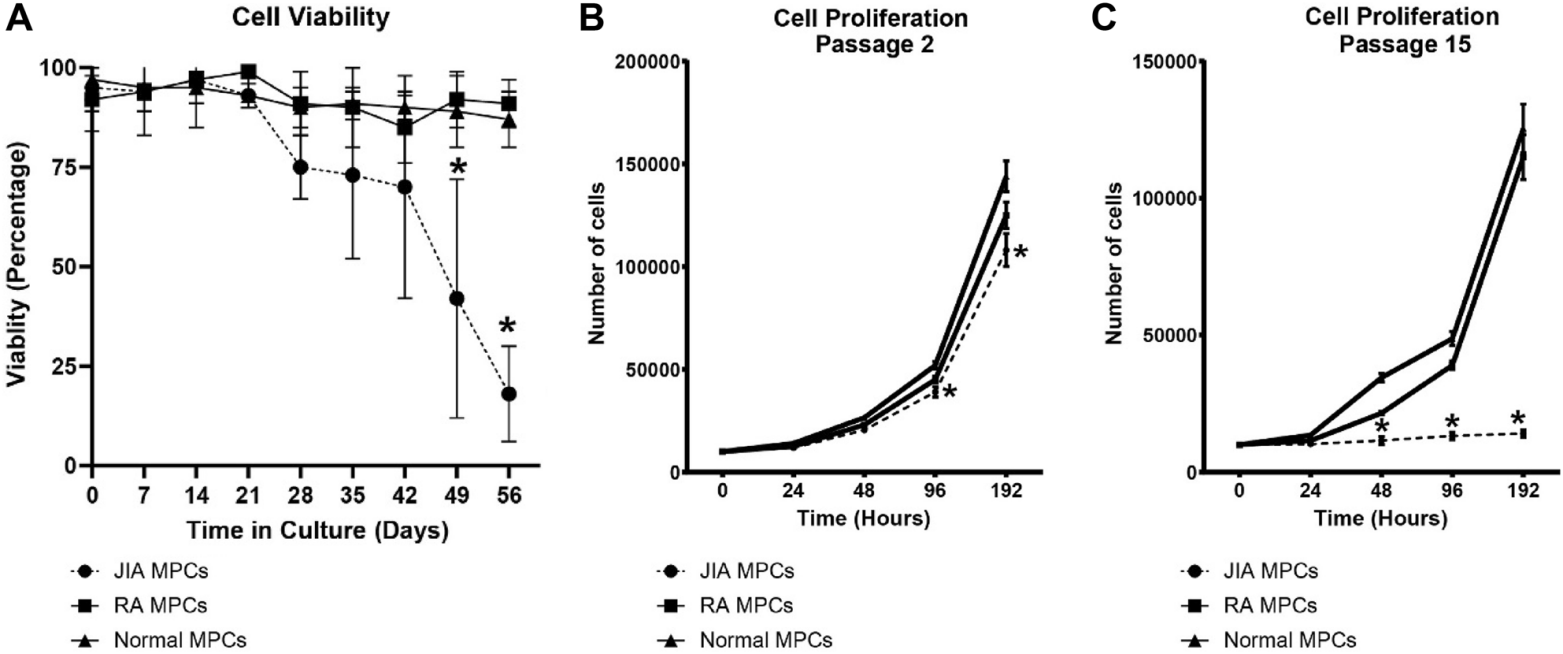
MPC viability and proliferation. Synovial MPCs from normal (n = 5), JIA (n = 19) and RA (n = 5) joints were assayed for cell viability (**A**) and cell proliferation at Passage 2 (**B**) and Passage 10 (**C**). *p < 0.05. Error bars represent mean + /- SD.

**Figure 2:**
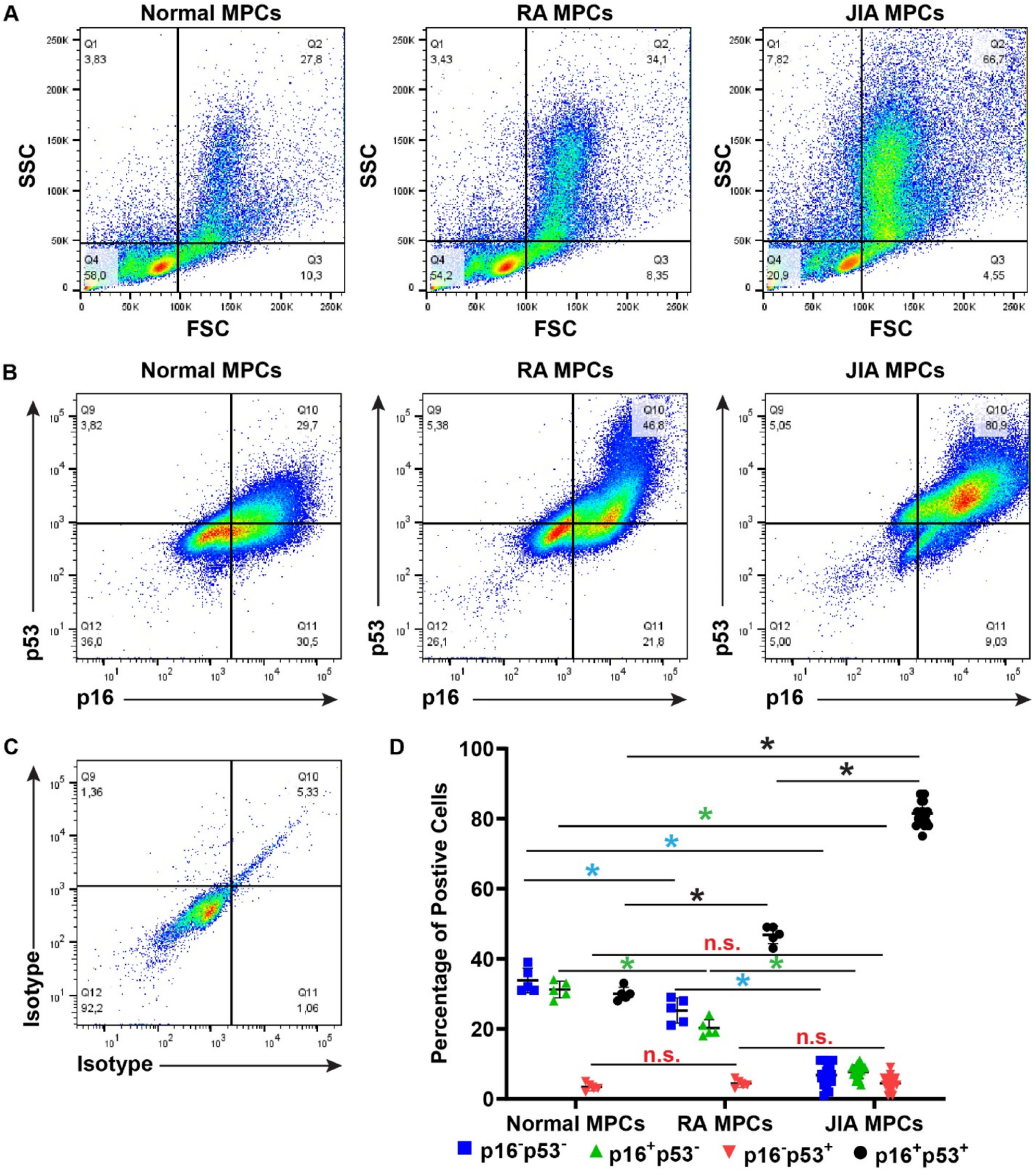
Expression of senescent marker p53 and p16 in MPCs at P10. Synovial MPCs from normal (n = 5), JIA (n = 19) and RA (n = 5) joints were assayed for morphological changes in cell shape/size using flow cytometry (**A**). MPCs from normal, RA and JIA populations were examined for expression of p53 and p16 (**B**). Isotype controls were used to developed thresholds for each marker (**C**). The p16^−^p53^−^, p16^+^p53^−^, p16^−^p53^+^ and p16^+^p53^+^ populations were quantified (**D**). *p < 0.05, n.s. = non-significant. Error bars represent mean + /- SD.

**Figure 3:**
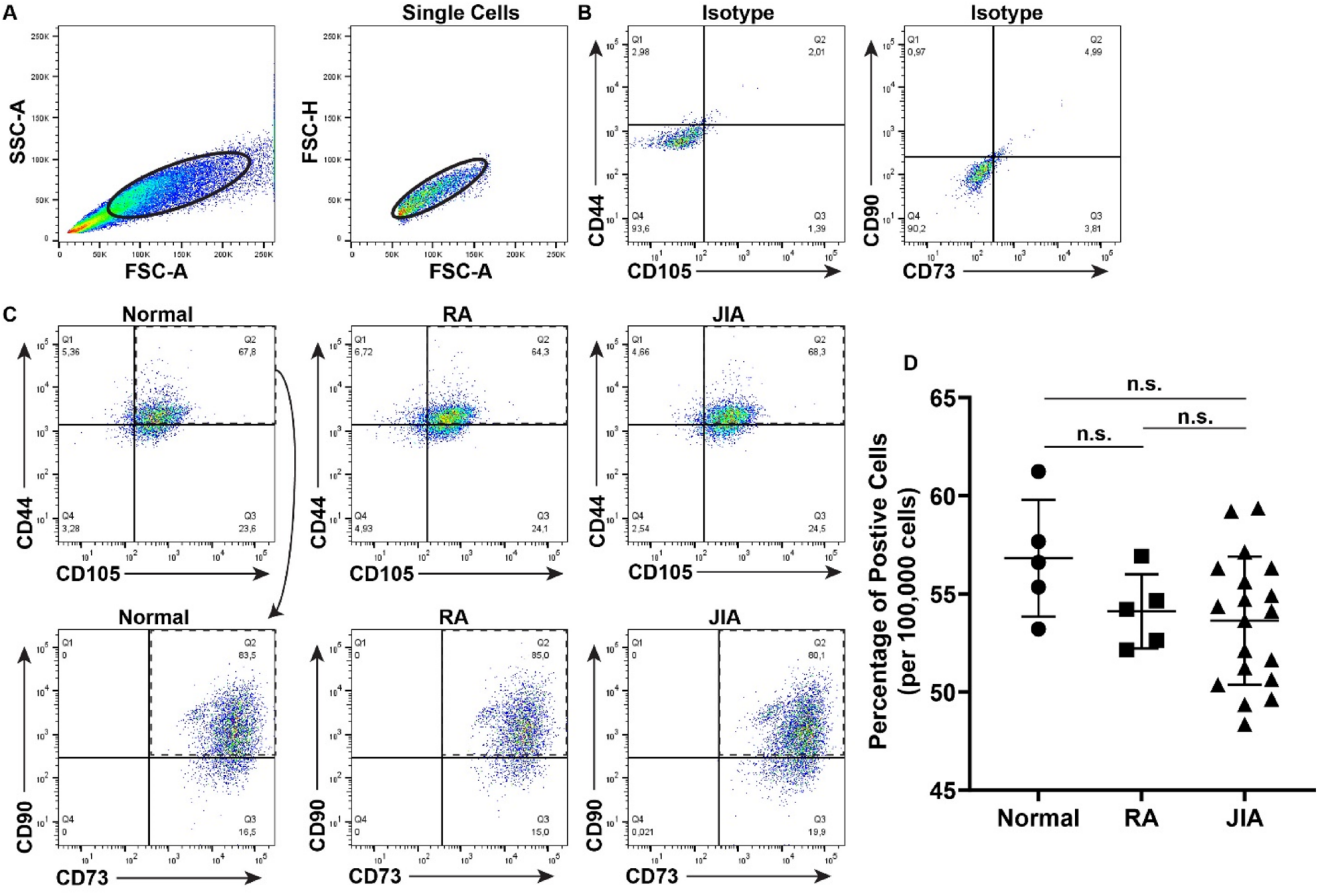
MPC abundance at P0**.** Synovial MPCs from normal (n = 5), JIA (n = 19) and RA (n = 5) joints were purified using FACS at P0. Gates were designed for the non-debris and single cell populations (**A**). Isotype controls were used to developed thresholds for each marker (**B**). The cells positive for CD44 and CD105 were then examined for expression of CD90 and CD73 (**C**). These CD105, CD90, CD73, CD44 positive MPCs were then quantified in each group (**D**). n.s. = non-significant. Error bars represent mean + /- SD.

Cell viability was assayed once per week for 8 weeks and while there were no differences in the viability between normal vs. RA MPCs (Fig. 1A), JIA MPCs showed a dramatic decrease in variability starting at approx. 28 days in culture, but this decrease only reached significance by 49 days in culture (Fig. 1A). By 56 days in culture, less than 20% of the JIA MPC population was viable. Based on this observation we performed a proliferation assay on early (passage 2) and late (passage 15) JIA, RA and normal MPCs. It was observed that JIA, normal and RA MPCs displayed similar cell proliferation rates at early passages, with only a slight decrease in JIA MPC proliferation (Fig. 1B), however, there was a dramatic decrease in JIA MPC proliferation rate at later passages (Fig. 1C). To determine if these JIA MPCs were undergoing cellular senescence, the cells were stained with p53 and p16 and assayed by flow cytometry at passage 10 (Fig. 2). Based on the SSC and FSC plots, a significant increase in cell size and granularity/density (SSC axis) could be observed in the JIA population which is consistent with hypertrophy and senescence (Fig. 2A, Figure S1)^42–47^. When we examined the expression of p53 and p16, we found significant increases in the p16^+^p53^+^ double positive population in the RA and JIA MPCs relative to normal MPCs (Fig. 2B,D). Furthermore, there was an increase in this p16^+^p53^+^ population in JIA MPCs relative to the RA MPCs with ~ 80% + of the JIA MPCs being double positive for p16 and p53 (Fig. 2B,D) Another potential explanation could be a difference in the relative abundance of MPCs in each patient group. Therefore, we compared the number of CD105^+^CD90^+^CD73^+^CD44^+^ cells in each sample (from normal, RA and JIA groups) at the time of FACS purification (P0)(Fig. 3A–D). There were no significant differences in this putative MPC population in any group at P0.

### Cell surface marker expression

**Figure 4:**
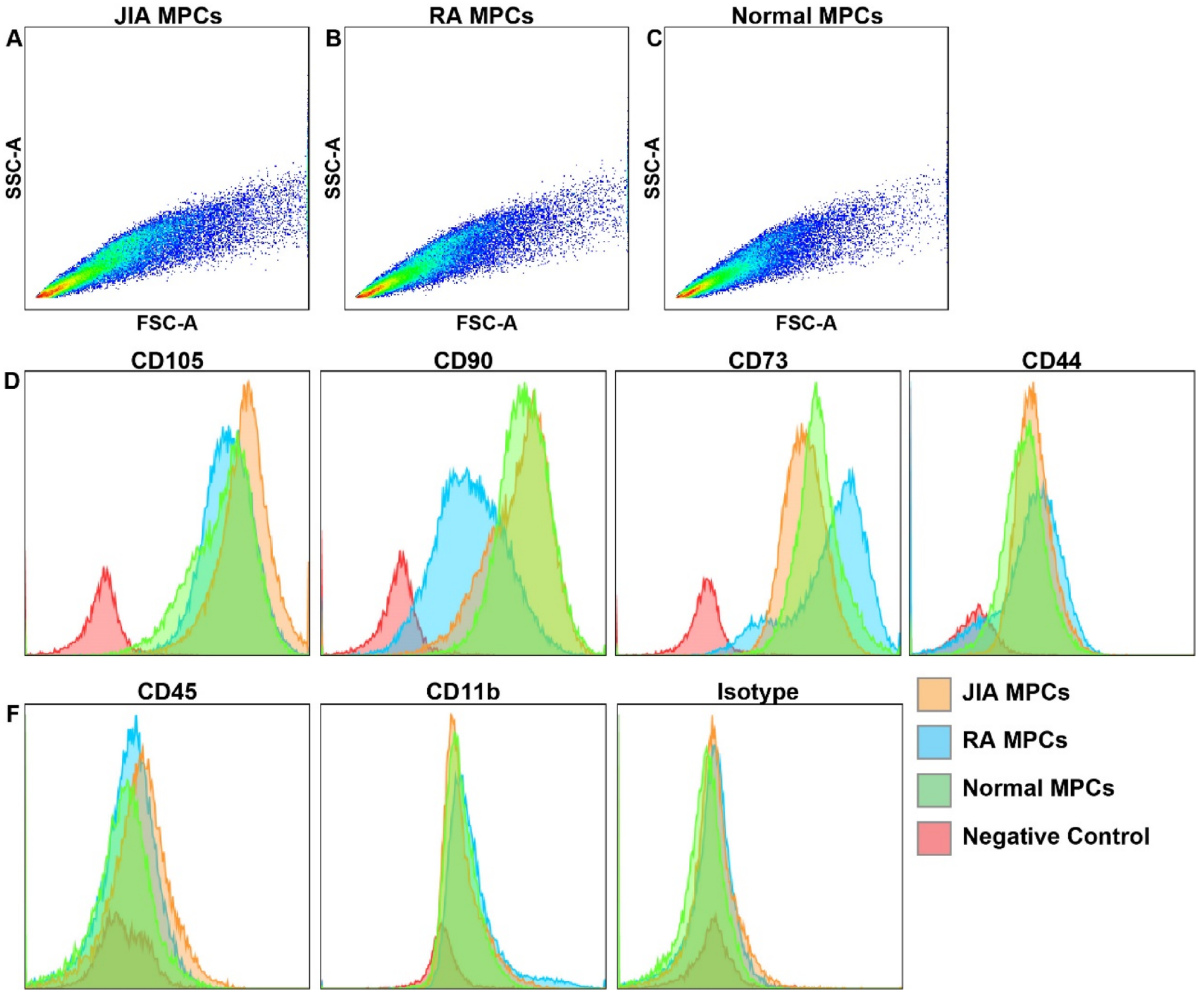
Cell surface marker profile of MPCs at P5. MPCs were assayed by flow cytometry for CD105, CD90, CD73, CD44, CD45, CD11b (**A**-**F**).

JIA, RA and normal MPCs were analyzed by flow cytometry to confirm that the cells retained expression of CD105, CD90, CD73, CD44 and lacked the expression of CD45 and CD11b after in vitro culture at P5 (Fig. 4A–F). MPCs from all three groups showed similar patterns of physical cell properties (diameter, complexity)(Fig. 4A–C) and cell surface marker expression (Fig. 4D–F). No significant differences in cell surfaces markers were identified when the results were quantified (Figure S2).

### Multi-lineage differentiation potential

**Figure 5:**
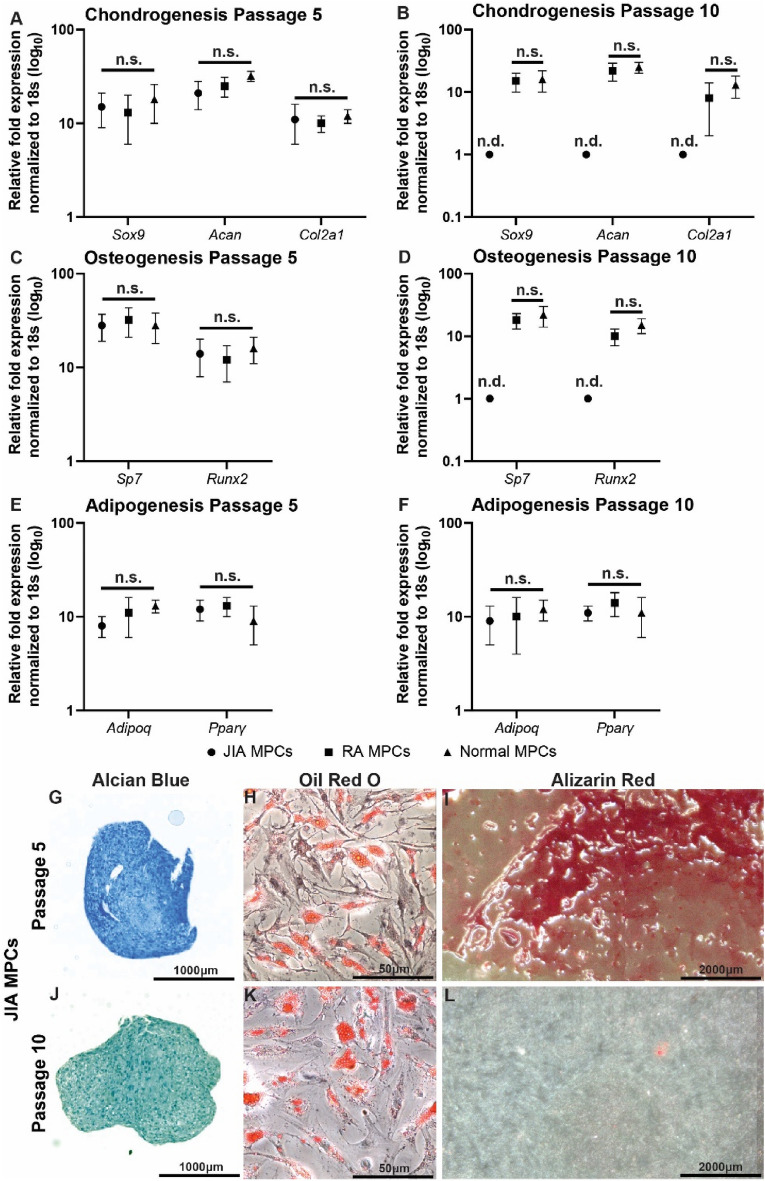
MPC differentiation potential. Synovial MPCs from normal (n = 5), JIA (n = 19) and RA (n = 5) joints were assayed for chondro-(**A**,**B**), osteo-(**C**,**D**) and adipogenic (**E**,**F**) differentiation capacity at passage 5 (**A**,**C**,**E**) and passage 10 (**B**,**D**,**F**) Differentiation was validated through the use of histological assessment of Alican Blue (**G**,**J**), Oil Red O (**H**,**K**) and Alizarin Red (**I**,**L**) in JIA MPCS at passage 5 (**G**-**I**) and passage 10 (**J**-**L**). n.s = not significant, n.d = not detected. Error bars represent mean + /- SD.

All MPCs were induced to undergo chondro, osteo and adipogenesis at passage 5 and passage 10 (Fig. 5). At passage 5, JIA, RA and normal MPCs displayed similar levels of chondrogenic (Fig. 5A), osteogenic (Fig. 5C) and adipogenic (Fig. 5E) markers post-differentiation and this was confirmed with histological staining (Fig. 5G–I – JIA MPCs shown as an example). However, at passage 10, JIA MPCs lost expression of chondrogenic (Fig. 5B) and osteogenic (Fig. 5D) markers, but retained expression of adipogenic (Fig. 5F) markers post-differentiation; which was confirmed by histological staining (Fig. 5 J–L).

### *Cartilage regeneration *in vivo

**Figure 6:**
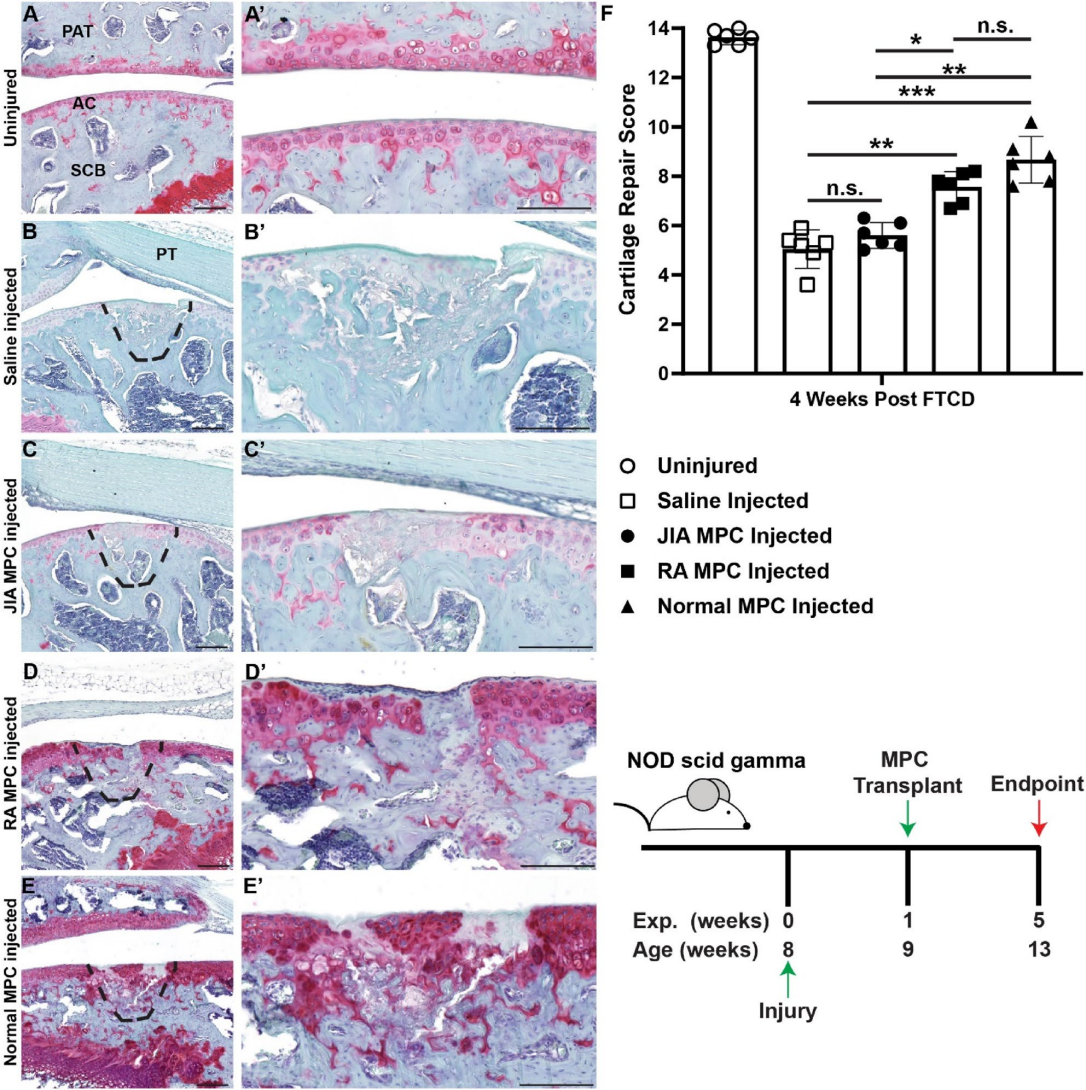
MPC mediated cartilage repair. Synovial MPCs from normal (n = 3), JIA (n = 3) and RA (n = 3) joints were injected into the injured joints of immunocompromised mice (n = 6 per group). Safranin O staining of uninjured (**A**,**A**’) and injured mice injected with saline (**B**,**B**’) or MPCs from JIA (**C**,**C**’), RA (**D**,**D**’) or normal (**E**,**E**) synovium. Cartilage repair across all groups was quantified (**F**). n.s = not significant, *p < 0.05, ** p < 0.01, *** p < 0.001. Error bars represent mean + /- SD. Scale bar equals 75 µm.

To determine if these MPCs (at P5) from different patient cohorts had the ability to effect cartilage regeneration in vivo, a xenotransplantation approach in an immunocompromised mouse injury model was employed. Uninjured cartilage demonstrated a robust Safranin O staining within the articular cartilage surface (Fig. 6A,A’), which was lost 4 weeks post-full thickness cartilage defect (FTCD) injury (Fig. 6B,B’). When JIA MPCs were transplanted into the FTCD site, only minor improvements in cartilage regeneration were observed (Fig. 6C,C’,F). This was in contrast to MPCs from RA or normal patients which demonstrated a robust regenerative response at 4 week post-FTCD (Fig. 6D,D’,E,E’,G), with normal MPCs being equal to RA MPCs in terms of cartilage regeneration (Fig. 6F).

*Contribution of MPCs *in vivo.

**Figure 7:**
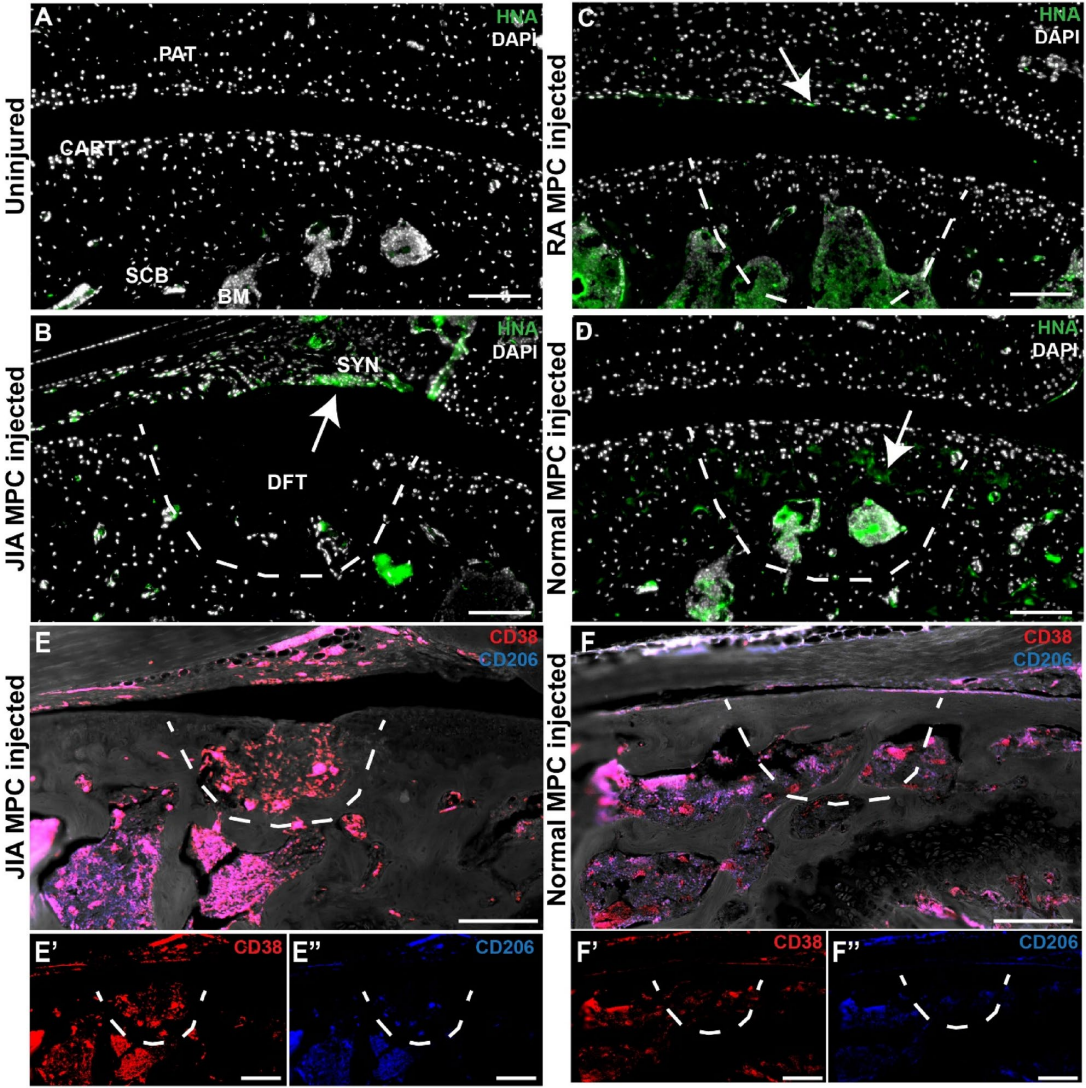
Transplanted human MPCs within the cartilage injury**.** Synovial MPCs from normal (n = 3), JIA (n = 3) and RA (n = 3) joints were injected into the injured joints of immunocompromised mice (n = 6 per group). Human Nuclear Antigen (HNA) staining was employed to detect JIA (**B**), RA (**C**) or normal (**D**) MPCs within the injury site. CD38^+^ (**E**’,**F**’) and CD206^+^ (**E”**,**F”**) macrophages within the injury site of mice injected with JIA (**E**) or normal (**F**) MPCs. Scale bar equals 75 µm.

We decided to examine if these xenotransplantated MPCs contributed to the formation of new joint tissue post-FTCD (Fig. 7). In uninjured and uninjected mice, no human nuclear antigen (HNA) staining was detected as expected (Fig. 7A). When JIA MPCs were injected into injured joints, few to no HNA^+^ cells were detected in the FTCD site, however, they were observed in the adjacent synovium (Fig. 7B). Although we saw increased cartilage regeneration in joints injected with RA MPCs, few to no HNA^+^ cells were found in the new cartilage but were again seen in the adjacent synovium (Fig. 7C). When normal MPCs were injected, HNA^+^ cells were observed within the regenerated cartilage and within the subchondral bone below the FTCD site (Fig. 7D).

### Response of endogenous macrophages to MPCs

Pro (CD38^+^) and anti-inflammatory (CD206^+^)^39^ macrophage populations were examined within the injury site post-MPC transplantation to determine how these MPCs impacted endogenous cell populations. When JIA MPCs were injected into the injury joint (Fig. 7E), the defect site was primarily comprised of CD38^+^ macrophages. In mice injected with normal MPCs (Fig. 7F), few macrophages were observed within the defect site, however, both CD38^+^ and CD206^+^ populations were observed adjacent to the injury site.

## Discussion

It is widely believed that JIA is not a single disorder, but a disease state that consists of a heterogeneous group of inflammatory diseases affecting joints with a prevalence of one in a thousand children^48^. While there are numerous front-line drugs that these children can be treated with^2,3^, there is a growing body of literature examining the use of mesenchymal stem/progenitor cells for the treatment of adult and childhood arthritis^8,10^. However, we still lack a clear understanding of how MPC populations are impacted by arthritic disease states and how this could influence treatment efficacy^25,30,49–51^. Therefore, in the current study, we examined the behaviour of synovial MPCs isolated from JIA patients in terms of proliferation, differentiation and cartilage repair potential in comparison to MPCs derived from RA patients and normal individuals.

Compared to the growing body of literature examining MPCs populations in the synovium and synovial fluid of adult populations with or without arthritis, there is a paucity of research on the corresponding cell populations within pediatric populations. In the current study, we found that JIA MPCs showed a dramatic decrease in proliferative capacity and differentiation capacity that was directly related to their time in culture which was not observed in adult MPC populations from normal or RA patients. The decreased proliferation capacity of synovial fluid MPCs from JIA has been previously examined and it was suggested that the inflammatory environment in the JIA joint could be a potential driver of this phenotype^52^. This hypothesis is likely at least partially correct since it has been demonstrated that cytokines and/or growth factors present in the arthritic (RA or OA) joint can dramatically impact the behvaiour of these resident MPC populations^25,53^. In the current study we also didn’t observe any meaningful differences in cell surface profile between JIA and normal/RA synovial fluid MPCs suggesting that whatever changes in the inflammatory milieu of the synovial fluid isn’t modifying this aspect of cell phenotype. We did however find that JIA MPCs lost nearly all chondrogenic and osteogenic differentiation capacity by passage 10 in culture while MPCs from normal and RA patients did not demonstrate loss in differentiation capacity with this duration of cell culture. While it is commonly accepted that primary MPCs populations will demonstrate a decrease in plasticity with extended culture^54^, in this study we only observed these changes in the MPC populations from JIA patients. This is interesting since a previous study has demonstrated a relationship between osteogenesis of synovial MPCs from JIA patients and the severity of their disease^55^. While all the JIA patient lines used in the current study behaved similar to each other, it would be of interest to sub-group based on disease severity and/or recruit additional patients with various disease activity/severity levels to see if the differentiation potential of the cells was directly linked to clinical phenotype. Furthermore, it would also be important to consider the severity of disease in addition to the type of disease modifying agent the patient is on, including their response of the patient to treatment. All of these factors may impact the behaviour of the cells including their ability to proliferate. Yet, it is important to note, that in our current study the adipogenic differentiation ability of the JIA MPCs was not impact by time in culture. This is also reflected in the literature, when a number of studies have shown that adipogenic capacity is typically the last (vs. chondro and osteo) lineage to be lost by primary human MPCs^56,57^. In regards to senescence, many groups including our own have shown that time in culture in addition to disease state and/or severity directly correlates with MPC senescence and by extension MPC potency^43,58,59^. There is also an expanding area of cell aging/senescence research that has focused on using non-labeling methods such as autofluorescence and/or cell size/granularity^44,46^. In the current study we employed traditional markers (p16, p53) in addition to cell size and granularity to determine senescence, however, it would also be advantageous to employ traditional methods such as β-galactosidase staining to achieve a more comprehensive picture of what is going on in these MPC populations^45^.

To examine if any of these differences in cell phenotype cold impact JIA MPCs ability to contribute to cartilage repair in vivo, we employed a mouse model of cartilage repair. In these experiments, we observed that JIA MPCs had little to no ability to effect cartilage repair in contrast to MPCs derived from normal and even RA synovial fluid. This result is particularly interesting since we used the cells at passage 5, when they still demonstrated multi-lineage differentiation potential and had yet not taken on a senescent phenotype. This suggests their lack of cartilage repair capacity is related to other phenotypic and/or molecular differences not identified in the current study and unbiased omics approaches (RNA-seq and/or proteomics) could be used to identify targets for further study. If we can identify why these JIA MPCs are deficient in this capacity, this information could not only be used to counteract this phenotype, but potentially also to increase the native ability of MPC population from other patient groups. Interesting, we did observe JIA MPCs post-transplant, however, they were localized to the synovium and not found within the defect/injury site. This suggests that these JIA MPCs were still able to integrate into the murine tissue post-transplant but maybe lacking the machinery to migrate to the site of injury and/or effect repair at a distance (paracrine/cell empowerment processes)^60^. We also found that that the transplantation of JIA vs. normal MPCs lead to different impacts on the endogenous macrophage populations. In the JIA injected mice, the unhealed defect site was enriched for CD38^+^ cells which have been associated with pro-inflammatory macrophages, while in the mice injected with normal MPCs, the injury site was relatively clear of CD38^+^ or CD206^+^ (anti-inflammatory macrophages). It remains unknow that if the macrophages are being directly regulated by the different MPCs populations, we and others have previously shown that macrophages populations are associated with articular cartilage regeneration vs. degeneration^39,61–63^. If it is the case the JIA synovial MPCs are fundamentally altered and unusable for regenerative medicine or tissue engineering applications that other allogenic or autologous sources would need to be considered ^64^.

## Conclusions

If and how MPCs from various disease/severity states regulate these outcomes will require further investigation, but the experiments conducted in the current study demonstrate a fundamental difference in the behavior of synovial fluid derived MPCs from JIA patients in comparison to corresponding normal and RA cell populations.

## Supplementary Information

Supplementary Information.

## Data Availability

The datasets used and/or analyzed during the current study are available from the corresponding author on reasonable request.
