# Supplementary material for: Synovial fluid mesenchymal progenitor cells from patients with juvenile idiopathic arthritis demonstrate limited self-renewal and chondrogenesis
Source: Sci Rep. 2022 Oct 3;12:16530. doi: 10.1038/s41598-022-20880-7 (PMC9530167; doi:10.1038/s41598-022-20880-7)
Supplement: Supplementary file 1 — Supplementary Information. [file 41598_2022_20880_MOESM1_ESM.docx]

**
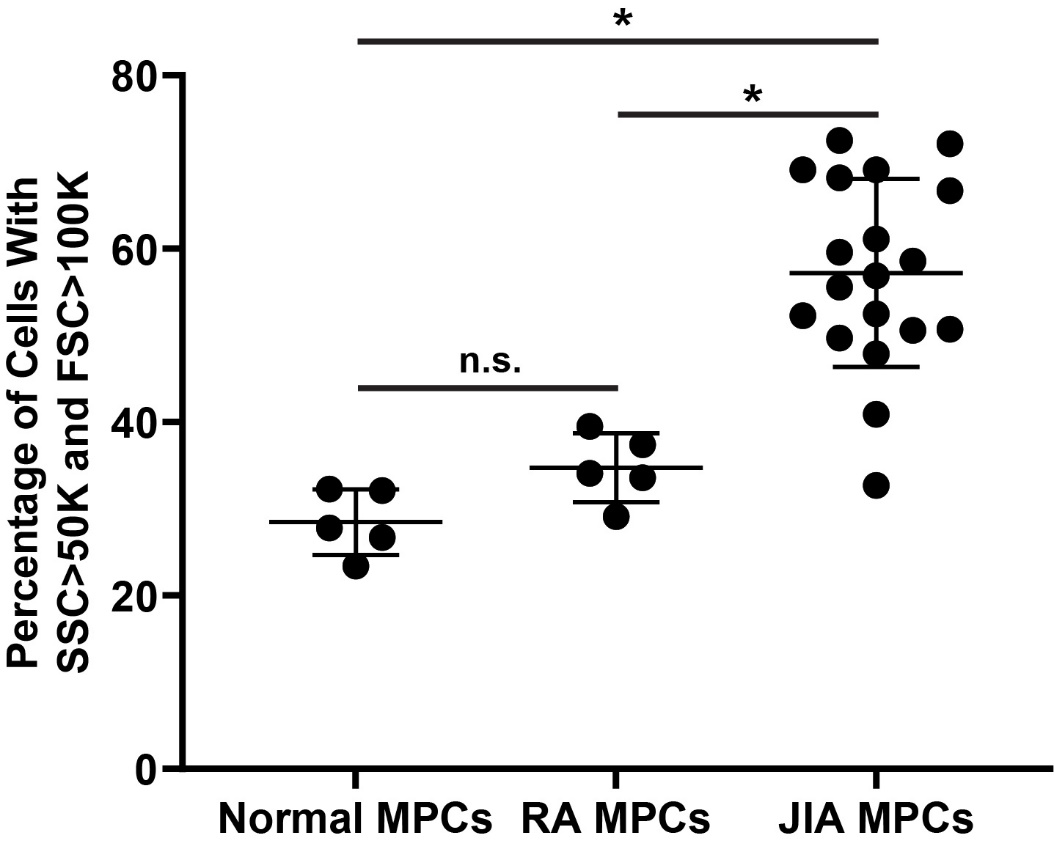
**

**Figure S1. Quantification of side scatter (SSC) and forward scatter (FSC) in MPCs.** Synovial MPCs from normal (n=5), JIA (n=19) and RA (n=5) joints were assayed for SSC and FSC using flow cytometry. A significant increase in SSC and FSC was observed in JIA MPCs vs. normal and RA MPCs. Error bars represent mean +/- SD. n.s. = not significant. * = p<0.05.

**
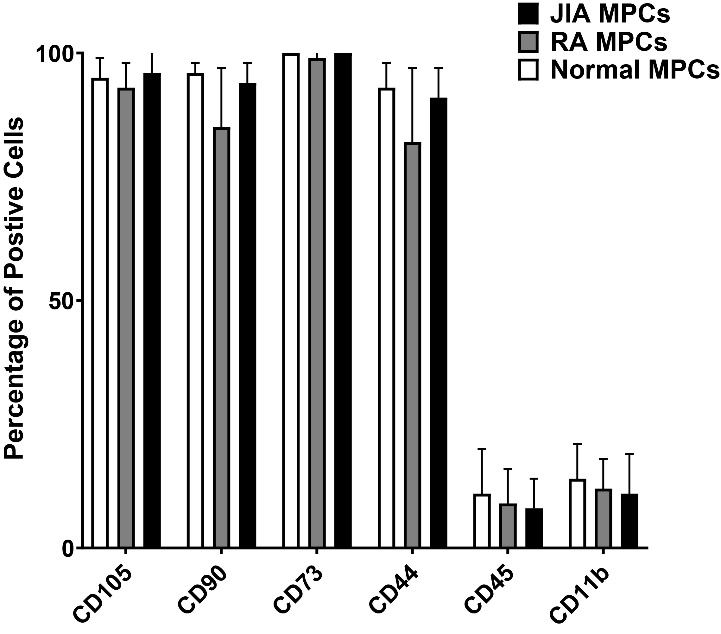
**

**Figure S2. Quantification of cell surfaces markers in MPCs.** Synovial MPCs from normal, JIA and RA joints were assayed for cell surfaces marker expression. No significant difference in any marker was observed. Error bars represent mean +/- SD.
